# Supplementary material for: Association of Skeletal Muscle and Adipose Tissue Distribution with Histologic Severity of Non-Alcoholic Fatty Liver
Source: Diagnostics (Basel). 2021 Jun 9;11(6):1061. doi: 10.3390/diagnostics11061061 (PMC8227703; doi:10.3390/diagnostics11061061)
Supplement: Supplementary file 1 [file diagnostics-11-01061-s001.zip › Table S1.docx]

Table S1. Adjusted odds ratio of high visceral adipose tissue index for fibrosis in patients with biopsy-proven non-alcoholic fatty liver disease

|  | Advanced liver fibrosis | |
| --- | --- | --- |
|  | OR (95% CI) | *P-*value |
| High VATI (yes vs. no) | | |
| Unadjusted | 7.17 (3.47-15.60) | <0.001 |
| Model 1 | 3.47 (1.48-8.40) | 0.005 |
| Model 2 | 3.52 (1.42-9.04) | 0.007 |
| Model 3 | 3.86 (1.43-11.13) | 0.009 |

OR, hazard ratio; CI, confidential interval

Model 1 was adjusted for age, gender.

Model 2 was adjusted for diabetes, hypertension, obesity inclusive of model 1.

Model 3 was adjusted for severe NASH, SMI, SATI inclusive of model 2.
