# Supplementary material for: Association of Skeletal Muscle and Adipose Tissue Distribution with Histologic Severity of Non-Alcoholic Fatty Liver
Source: Diagnostics (Basel). 2021 Jun 9;11(6):1061. doi: 10.3390/diagnostics11061061 (PMC8227703; doi:10.3390/diagnostics11061061)
Supplement: Supplementary file 1 [file diagnostics-11-01061-s001.zip › Table S2.docx]

Table S2. Sex differences in baseline characteristics

| Variable | Male  n = 86 (48.3 %) | Female  n = 92 (51.7 %) | *P* value |
| --- | --- | --- | --- |
| Age, years | 42.0 [29.0-60.0] | 57.5 [49.5-64.5] | <0.001 |
| Body mass index, kg/m^2^ | 27.3 [25.4-30.1] | 26.3 [24.0-28.8] | 0.020 |
| Comorbidities, n (%) |  |  |  |
| Obesity | 67 (77.9) | 56 (60.9) | 0.022 |
| Type 2 diabetes | 19 (22.1) | 35 (38.9) | 0.024 |
| Hypertension | 27 (31.4) | 33 (36.7) | 0.563 |
| Muscle and fat distribution based on CT |  |  |  |
| SMI, cm^2^/m^2^ | 57.8 [52.5-63.6] | 46.8 [43.2-50.7] | <0.001 |
| SATI, cm^2^/m^2^ | 56.6 [43.6-80.4] | 67.8 [55.3-91.5] | 0.001 |
| VATI, cm^2^/m^2^ | 66.9 [51.2-91.6] | 65.4 [50.0-78.3] | 0.689 |
| SMI, sex-specific COVs, n (%) | 18 (20.9) | 5 (5.4) | 0.004 |
| SATI, sex-specific COVs, n (%) | 34 (39.5) | 37 (40.2) | 1.000 |
| VATI, sex-specific COVs, n (%) | 19 (22.1) | 50 (54.3) | <0.001 |
| Biopsy profiles |  |  |  |
| NAFLD activity score | 4.0 [3.0-5.0] | 4.0 [3.0-5.0] | 0.101 |
| Severe NASH, n (%) | 25 (29.1) | 36 (39.1) | 0.209 |
| Advanced fibrosis, n (%) | 12 (14.0) | 35 (38.0) | 0.001 |

Values are expressed as median (interquartile range [IQR]) or n (%).

CT, computed tomography; SMI, skeletal muscle index; SATI, subcutaneous adipose tissue index; VATI, visceral adipose tissue index; COV, cut-off value; NAFLD, non-alcoholic fatty liver disease; NASH, non-alcoholic steatohepatitis.
