# Supplementary figures and images for: Association of Skeletal Muscle and Adipose Tissue Distribution with Histologic Severity of Non-Alcoholic Fatty Liver
Source: Diagnostics (Basel). 2021 Jun 9;11(6):1061. doi: 10.3390/diagnostics11061061 (PMC8227703; doi:10.3390/diagnostics11061061)

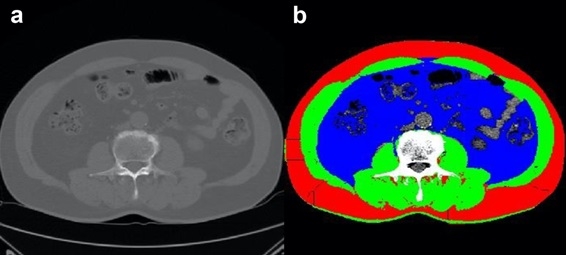

Supplement: Supplementary file 1 [file diagnostics-11-01061-s001.zip › Figure S1.jpg]

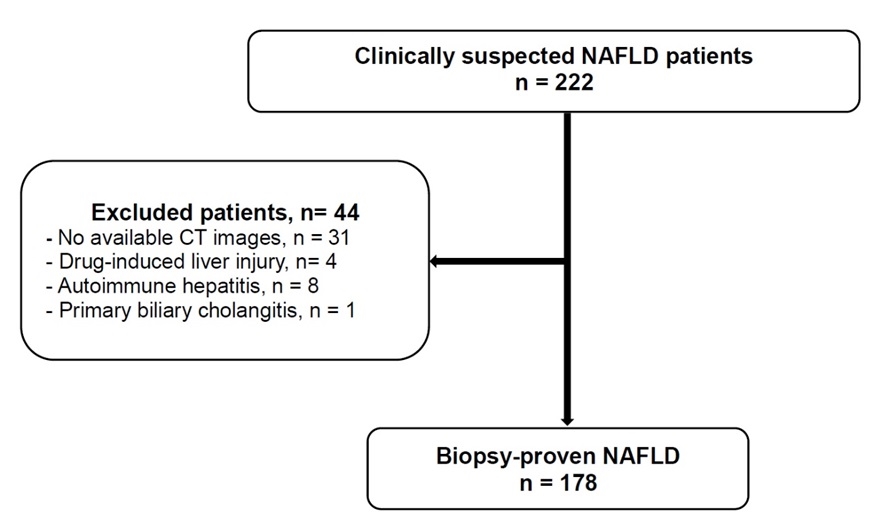

Supplement: Supplementary file 1 [file diagnostics-11-01061-s001.zip › Figure S2.jpg]

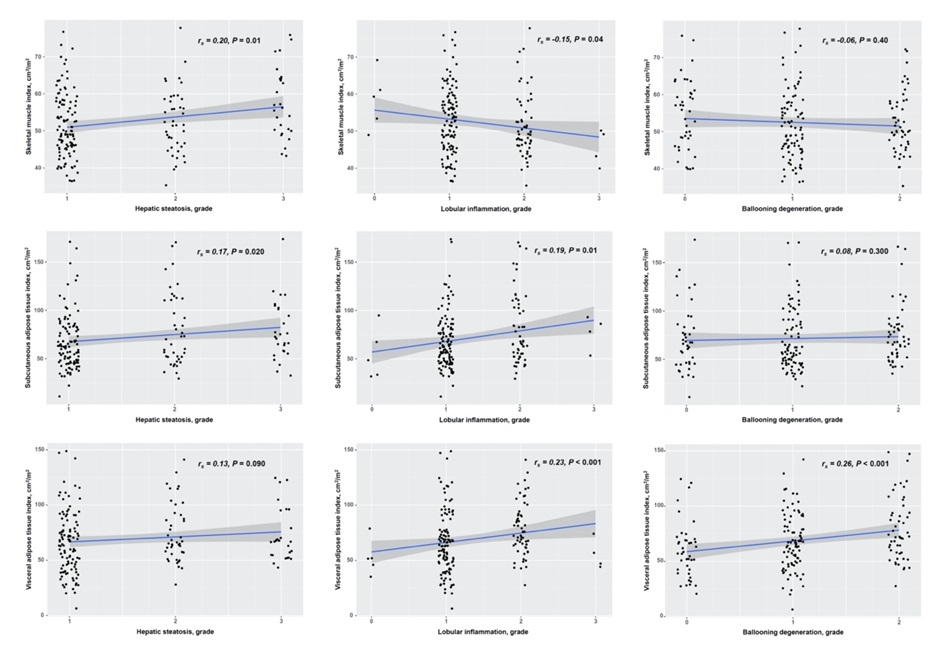

Supplement: Supplementary file 1 [file diagnostics-11-01061-s001.zip › Figure S3.jpg]

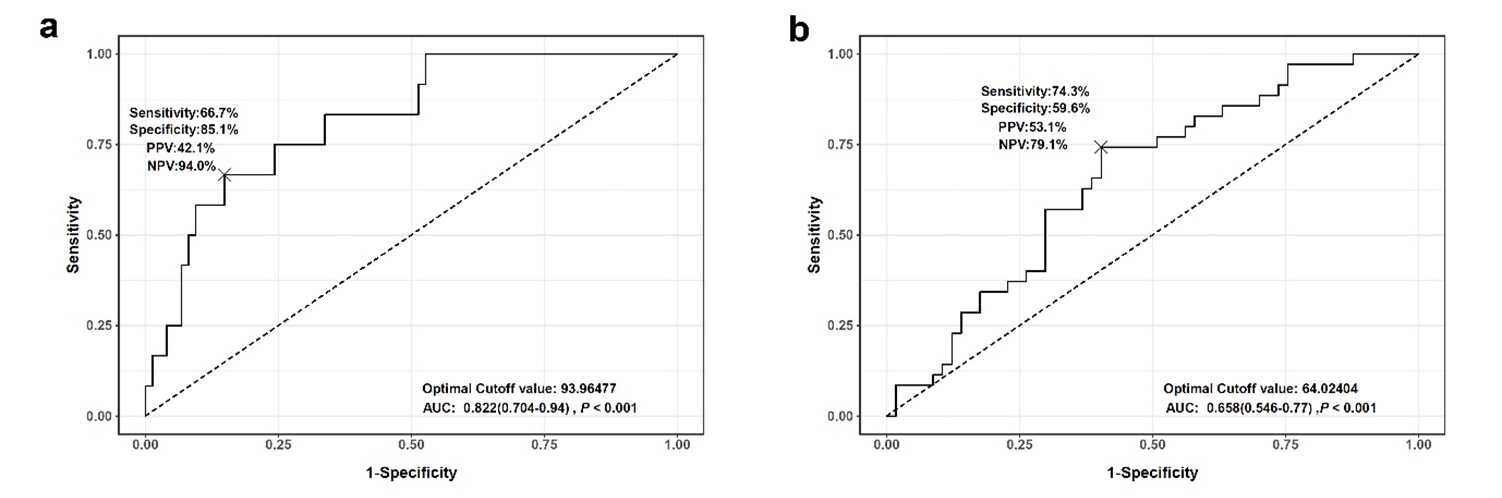

Supplement: Supplementary file 1 [file diagnostics-11-01061-s001.zip › Figure S4.jpg]

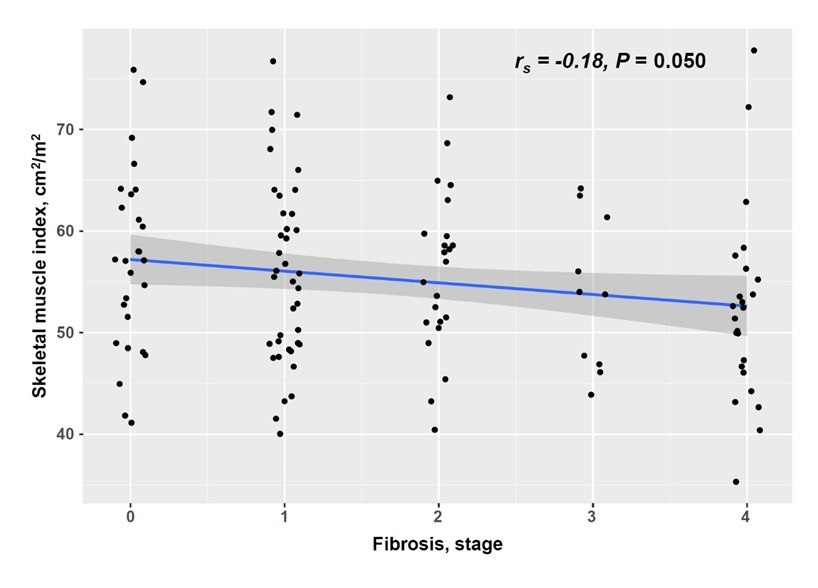

Supplement: Supplementary file 1 [file diagnostics-11-01061-s001.zip › Figure S5.jpg]

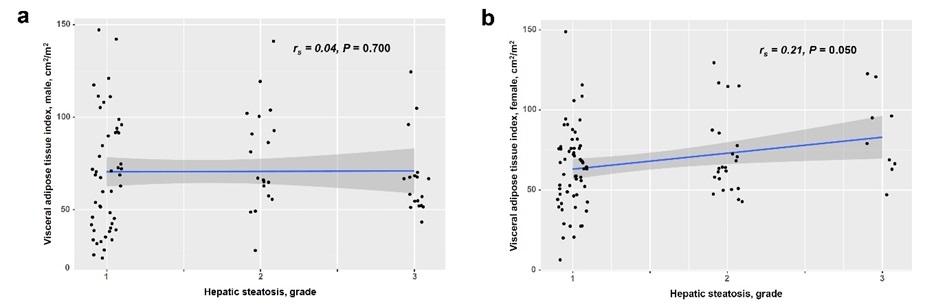

Supplement: Supplementary file 1 [file diagnostics-11-01061-s001.zip › Figure S6.jpg]
